# The incidence and severity of open gingival embrasures in adults treated with clear aligners and fixed appliances: a retrospective cohort study

**DOI:** 10.1186/s13005-023-00375-0

**Published:** 2023-07-17

**Authors:** Tianrui Yang, Lishan Jiang, Weiman Sun, Meng Zhu, Ke Jiang, Houxuan Li, Lang Lei

**Affiliations:** 1 Nanjing Stomatological Hospital, Affiliated Hospital of Medical School, Nanjing University, Nanjing, Jiangsu China; 2 Department of Periodontology, Nanjing Stomatological Hospital, Affiliated Hospital of Medical School, Nanjing University, 30 Zhongyang Road, Nanjing, Jiangsu 210008 China; 3 Department of Orthodontics, Nanjing Stomatological Hospital, Affiliated Hospital of Medical School, Nanjing University, 30 Zhongyang Road, Nanjing, Jiangsu 210008 China

**Keywords:** Open gingival embrasure, Clear aligner, Fixed appliance, Orthodontic treatment

## Abstract

**Background:**

To evaluate the incidence and severity of open gingival embrasures (OGEs) in adult patients treated with clear aligners and fixed appliances.

**Methods:**

Two hundred non-extraction adult subjects with less than 5 mm of crowding (mean age, 24.6 ± 3.8 years) were enrolled in this retrospective study. The subjects were divided into the clear aligner (*n* = 100) and fixed appliance group (*n* = 100). The intraoral photographs were utilized to determine the incidence of OGEs in the upper arch between maxillary central incisors, as well as the lower arch between mandibular central incisors. Crown overlap, crown shape, posttreatment root angulation, the distance from the interproximal contact point (ICP) to the alveolar bone crest (ABC) after treatment and interproximal enamel reduction (IPR) were determined in the two groups.

**Results:**

The incidence of OGEs between maxillary and mandibular central incisors after orthodontic treatment was 35.0% and 38.0% in the clear aligner group, respectively, significantly higher than that (18.0% and 24.0%) in the fixed appliance group (*P* < 0.05). The average area of an OGE after clear aligner treatment was larger both in the maxilla (0.16 ± 0.12mm^2^) and mandible (0.21 ± 0.24mm^2^) compared with that (0.05 ± 0.03mm^2^ and 0.05 ± 0.06mm^2^) after fixed appliance treatment (*P* < 0.05). No difference was found regarding pretreatment crown overlap, crown shape, treatment duration, posttreatment root angulation, amount and distribution of IPR and the distance from ICP to ABC.

**Conclusions:**

The incidence and severity of OGEs were higher in adults treated with clear aligners. Clinicians should be aware of the risk of OGEs during treatment with clear aligners.

## Background

The incidence of open gingival embrasures (OGEs), which are also called as black triangles, is a result of incomplete filling of the space between adjacent teeth by interdental papilla. An OGE is an undesirable side effect during orthodontic treatment, not uncommon in adult patients. The incidence of OGEs between maxillary central incisors among adult orthodontic patients was 22% ~ 41.9% [1, 2]. It is a failure to meet esthetic demands as well as a risk of periodontal health because of plaque retention [3].

Several factors contribute to the occurrence of OGEs. Irregularity in the incisor region is one of the most important risk factors. Two-thirds of adult orthodontic patients with severely crowded central incisors had OGEs after treatment [3]. The root angulation, the distance from the alveolar bone to interproximal contact position, and crown form also contribute to the presence of OGEs [1–3]. Moreover, extraction of lower incisors leads to a high incidence of OGEs in an early clinical evaluation [4]. The severity of OGEs was also pointed out to be associated with IPR in a study recently [5].

The popularity of clear aligners has been increasing in the adults seeking orthodontic treatment. Clear aligners are more favorable in aesthetics than traditional labially-placed fixed appliances [6]. As a kind of removable appliance, clear aligner is more physically advantageous for the oral hygiene than the fixed buccal and lingual appliances. Less plaque index and superior periodontal health have been reported in clear aligners than fixed appliances [7, 8]. However, clear aligners cover the entire dental arch for 22 h every day as recommended by most manufacturers and orthodontists, extending well below the contact point for good retention. Such gingival extension may change the anatomical features for the remodeling of the gingival papilla, occupying the interproximal space with aligner materials rather than gingiva. Therefore, the difference in the oral hygiene and anatomic features may affect the incidence of OGEs, which is of great significance for adult patients.

Most previous researches focus on the anatomic characters of factors, such as crown shape, root angulation, severity of crowding when assessing OGEs. However, currently no clinical study has been designed to evaluate whether the type of appliances may affect the occurrence of OGEs. Therefore, the purpose of this study was to evaluate and compare the incidence and severity of OGEs in adult patients treated with clear aligners and fixed appliances.

## Methods

### Subjects

This retrospective cohort study was approved by the Ethical Committee of Nanjing Stomatological Hospital, Medical School of Nanjing University (approval no. KY-2020NL-064). Subjects were enrolled from a pool of patients finishing orthodontic treatment between March 2016 and July 2021 at the Department of Orthodontics, Nanjing Stomatological Hospital, Nanjing University. All the patients were above 18 years old at the beginning of the orthodontic treatment. The subjects were diagnosed as periodontally healthy or minor periodontitis (stage I, 2017 new classification by the American Academy of Periodontology (AAP) and the European Federation of Periodontology (EFP)) and no tooth extraction except the third molars were included in the treatment. Intraoral photographs, panoramic radiographs and digital models before and after treatment were complete for all patients. All patients read and signed an informed consent to participate in this study.

These patients were excluded if: (1) they have moderate or severe periodontal diseases; (2) they had a history of orthodontic treatment; (3) there was a disagreement of classifications among 5 raters; (4) they lacked complete intraoral photographs, panoramic radiographs or digital models before and after treatment; (5) their records were in low quality that may influence the measurement. The patients were finished fully by clear aligners (Invisalign, Align Technology, California, USA) (*n* = 100) or passive-ligation brackets (Damon Q, Ormco, California, USA) (*n* = 100). All the patients received initial nonsurgical periodontal therapy including scaling and root planing, supportive periodontal therapy and oral hygiene instructions throughout the orthodontic treatment to help obtain periodontal health. Patients were referred to periodontal department once obvious periodontal inflammation was present, and no flap surgery was performed in the incisor region in all the subjects.

### Measurement of crown overlap and rotation

**Fig. 1:**
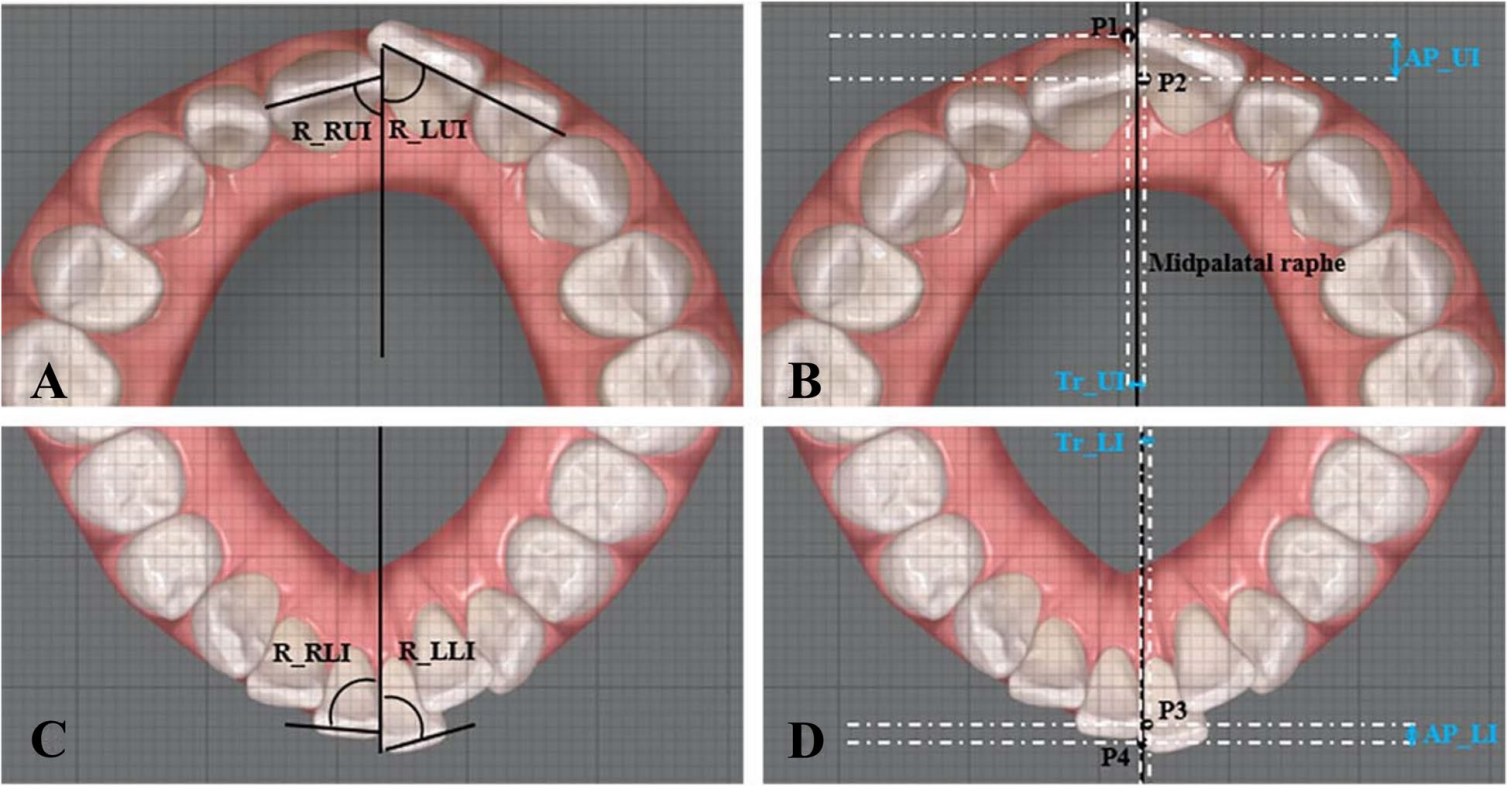
Measurement of central incisor rotation and overlap in the maxilla and mandible before treatment. The side length of each small square is 1 mm. **A** R_RUI indicates rotation of right upper incisor. R_LUI indicates rotation of left upper incisor. **B** Tr_UI indicates transverse overlap of upper incisors. AP_UI indicates antero-posterior overlap of upper incisors. P1 and P2 indicate mesial points of maxillary central incisor edges. **C** R_RLI indicates rotation of right lower incisor. R_LLI indicates rotation of left lower incisor. **D** Tr_LI indicates transverse overlap of lower incisors. AP_LI indicates antero-posterior overlap of lower incisors. P3 and P4 indicate mesial points of mandibular central incisor edges

Pretreatment photographs of maxillary and mandibular digital models in ClinCheck (Align Technology, California, USA) and Dolphin Imaging Software (Dolphin Imaging & Management Solutions, Chatsworth, USA) were provided with scales. The images were imported for analysis into the ImageJ program (National Institute of Health, USA). Reference lines were constructed as the midpalatal raphe in the maxilla and the perpendicular bisector of the line passing mesial contact points of bilateral first molars in the mandible. The rotation was defined as the angle formed by the central incisor edge and reference lines (Fig. 1A, C). The antero-posterior and transverse overlap were measured as the distance between the mesial points of central incisor edges parallel and perpendicular to the reference lines respectively (Fig. 1B, D).

### Measurement of root angulation and the distance from interproximal contact point (ICP) to the alveolar bone crest (ABC)

**Fig. 2:**
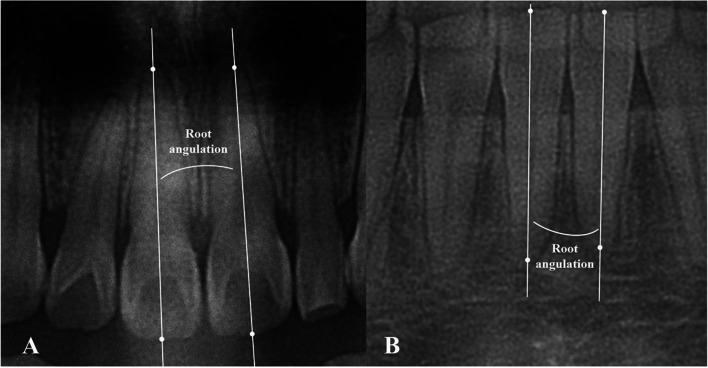
Measurement of root angulation on panoramic radiographs after treatment. **A** Root angulation of maxillary central incisors. **B** Root angulation of mandibular central incisors

The posttreatment root angulation and distance from ICP to ABC were measured on panoramic radiographs by Image J. The root angulation was defined as the angle between long axes of adjacent central incisors (Fig. 2). The value was zero if the roots were parallel. The value would be positive for divergent roots while negative otherwise.

**Fig. 3:**
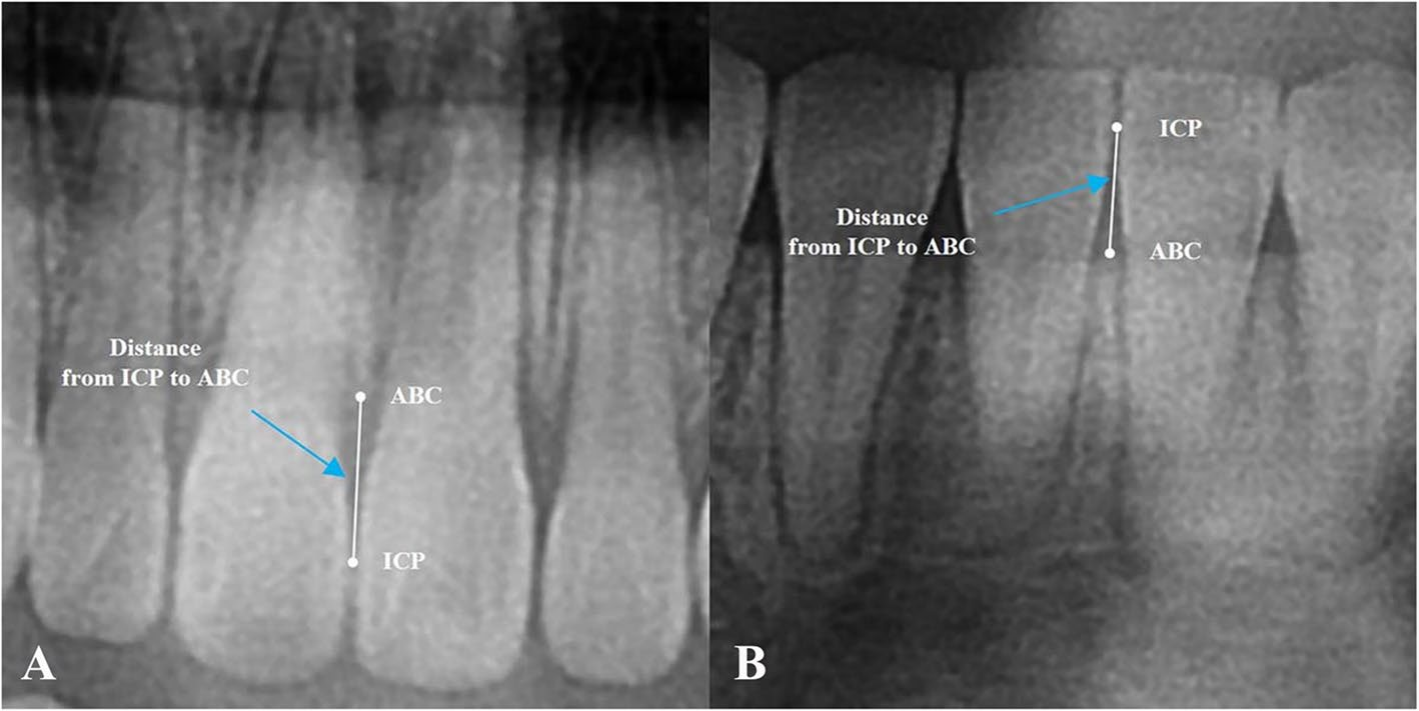
Measurement of the distance from ICP to ABC. **A** Distance from the ICP to ABC between the central incisors in the maxilla. **B** Distance from the ICP to ABC between the central incisors in the mandible

The ICP was defined as the most gingival point of the interproximal contact surface between the central incisors. The ABC was the most coronal area of the crestal bone. And the distance from ICP to ABC was measured perpendicular to the alveolar crest (Fig. 3).

### Measurement of crown morphology and interproximal enamel reduction (IPR)

**Fig. 4:**
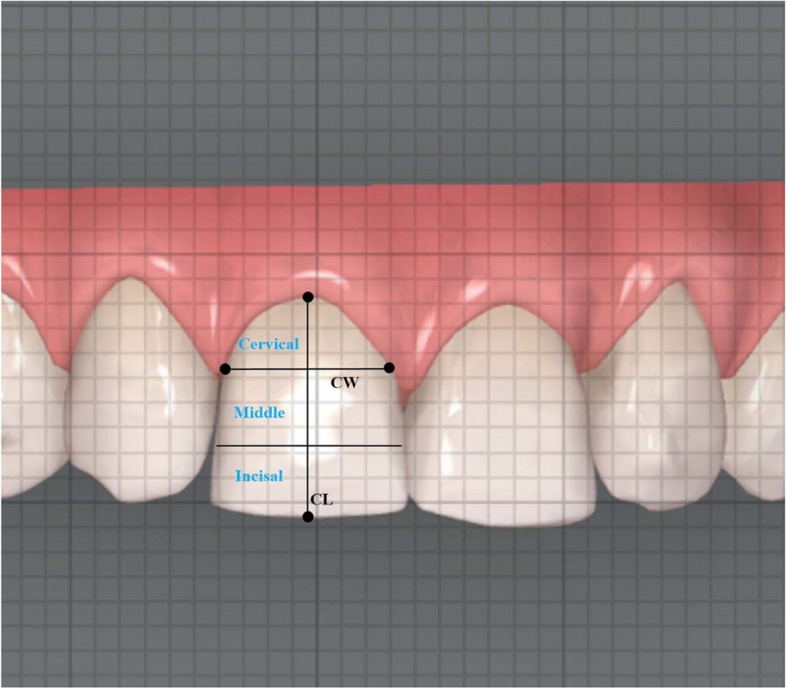
Measurement of the crown ratio of central incisors. CW indicates crown width. CL indicates crown length

The crown morphology of the central incisor was assessed on posttreatment digital models, represented by the ratio of crown width (CW) and crown length (CL). The crown length (CL) was the distance from gingival zenith to the middle of incisal edge of the crown. Then, the crown length was evenly divided into three parts: incisal, middle and cervical. The crown width (CW) was measured as the mesio-distal distance at the borderline between the middle 1/3 and cervical 1/3 as described by Olsson et al. [9] (Fig. 4).

The data of interproximal enamel reduction (IPR) were collected in the patients’ records.

### Incidence and severity of OGEs

**Fig. 5:**
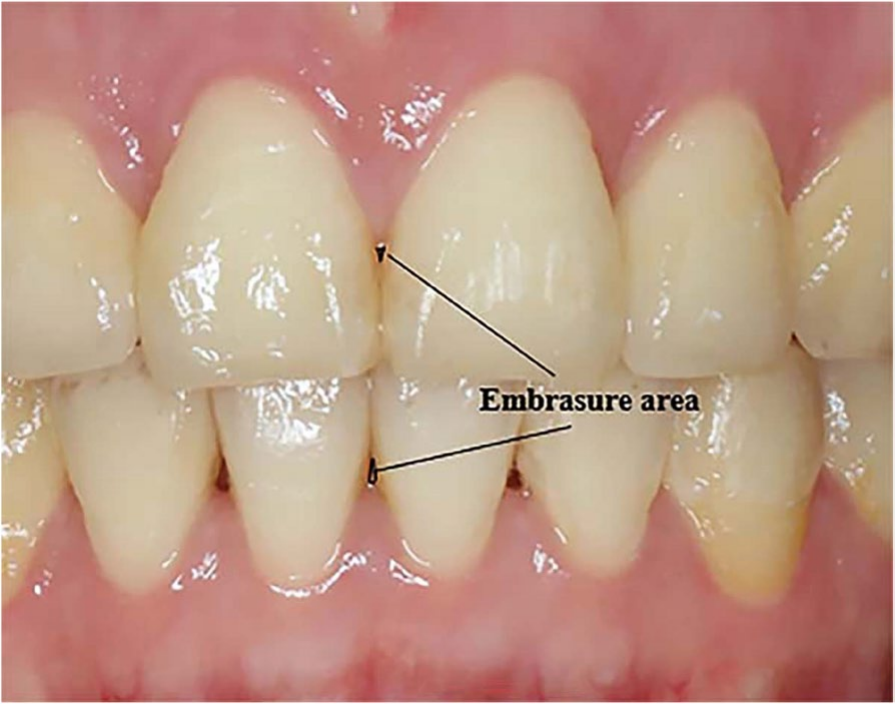
Measurement of areas of open gingival embrasures

The posttreatment intraoral photographs were evaluated by three orthodontists and two periodontists individually to determine the incidence of OGEs between the maxillary and mandibular central incisors. The 5 raters determined the existence of OGEs and classified them according to a system developed by Nordland and Tarnow [10]. Patients were included only when at least 4 of 5 raters made the same judgement. Subjects with OGEs were all assigned to the Class 1 group consequently. Thus, the area of OGEs was measured to further identify the severity. The actual CL was measured on the digital model. The magnification factor (MF) was defined as the ratio of CL in the intraoral photograph and the actual CL. In the photograph, the height of an OGE was the distance between the uppermost margin of interdental papilla and the contact point of central incisors. The width of an OGE was the distance at the level of the uppermost margin of interdental papilla (Fig. 5). Therefore, the actual width and height of an OGE was calculated as the measurement in the photograph divided by MF, respectively.

### Statistical analysis

The IBM SPSS Statistics 26 (International Business Machines Corp., Chicago, IL, USA) was used for statistical analysis. The level of significance was set at *P*< 0.05. The sample size required for this study was estimated by G*Power 3.1.9.7 (Franz Faul, Universität). The determination was based on previous estimates of incidence of OGEs between central incisors in patients with fixed appliances [2]. A total of 200 patients (100 in each group) were required to determine a significant difference in incidence of OGEs with a significance level of 0.05 and a power of 0.8 using chi-square tests.

One examiner performed all measurements. The evaluators were blinded to the purpose of the study. The intraoral photographs, panoramic radiographs and digital models, patients’ identity and grouping were concealed to minimize the observer bias. Every measurement was performed three times and the average value was taken as the final result to reduce measurement error. The intraclass correlation coefficient was 0.992 (*P* < 0.001), indicating high reliability. The kappa statistic of interrater category ratings was 0.66 (*P* < 0.001), indicating substantial agreement.

The difference in incidence, gender and distribution of IPR between the two groups were analyzed by Pearson’s chi-squared test. The Wilcoxon test was used to compare the embrasure areas between the two groups. The independent t-tests were used to analyze pretreatment crown overlap, crown shape, treatment duration, age and posttreatment root angulation. The distance from ICP to ABC and the amount of IPR were analyzed by the independent t-test as well.

## Results

### Baseline characteristics of subjects

The gender distribution was similar between the two groups (*P* > 0.05; Table 1). Although patients in the clear aligner were older and experienced longer treatment duration, the differences were not statistically significant.

**Table 1:** Baseline characteristics of subjects in the two groups

	Clear aligners (*n* = 100)	Fixed appliances (*n* = 100)	*P* value
Age (Mean ± SD, yrs)	25.13 ± 4.11	24.14 ± 3.51	0.068
Treatment time (Mean ± SD, month)	26.39 ± 7.67	25.72 ± 9.71	0.589
Gender (N, %)			0.451
Male	15 (15.0%)	19 (19.0%)	
Female	85 (85.0%)	81 (81.0%)	

Independent t-tests were used to compare age and treatment time between the clear aligner and fixed appliance group. A chi-square test was used to compare gender distribution between the two groups

*SD* Standard deviation

### Overlap and rotation of central incisors

The rotation was calculated as the absolute value of the measured angle subtracted from 90°. Table 2 showed that patients treated with fixed appliances exhibited greater rotation before treatment than those treated with clear aligners in the maxilla. The result was opposite for the mandibular left central incisor. However, no statistically significant differences were found (*P* > 0.05) on overlap and rotation between the two groups.

**Table 2:** Measurement of incisor overlap and rotation before treatment

Measurement (Mean ± SD)	Maxilla			Mandible		
	Clear aligners	Fixed appliances	*P* value	Clear aligners	Fixed appliances	*P* value
Right incisor rotation, degrees	8.66 ± 6.78	10.42 ± 11.29	0.184	10.36 ± 11.99	10.44 ± 9.03	0.960
Left incisor rotation, degrees	7.92 ± 7.10	8.00 ± 7.56	0.934	11.96 ± 12.87	9.63 ± 8.69	0.135
Transverse overlap, mm	0.11 ± 0.42	0.10 ± 0.20	0.907	0.10 ± 0.15	0.13 ± 0.21	0.213
Anterior–posterior overlap, mm	0.35 ± 0.40	0.37 ± 0.57	0.809	0.52 ± 0.63	0.39 ± 0.52	0.120

Independent t-tests were used to compare pretreatment variables between the clear aligner and fixed appliance group

*SD* Standard deviation

### Root angulation, crown shape, distance from ICP to ABC and IPR

As for the posttreatment root angulation and crown morphology, the differences between the two groups were not statistically significant (*P* > 0.05; Table 3). Although greater average value was observed regarding the distance from ICP to ABC of central incisors in patients treated with clear aligners, the differences were not statistically significant. The number of IPR sites was 28 between maxillary central incisors, and 31 between mandibular central incisors in the clear aligner group, while the number was 26 and 29 in the fixed appliance group. No difference was found regarding the amount and distribution of IPR between two groups (*P* > 0.05; Table 3).

**Table 3:** Measurement of root angulation, crown shape, distance from ICP to ABC and IPR

Measurement (Mean ± SD)	Maxilla			Mandible		
	Clear aligners	Fixed appliances	*P* value	Clear aligners	Fixed appliances	*P* value
Root angulation, degrees	0.01 ± 3.46	0.17 ± 3.26	0.137	0.51 ± 3.49	0.26 ± 4.30	0.655
Crown morphology	0.79 ± 0.07	0.80 ± 0.06	0.111	0.68 ± 0.07	0.70 ± 0.06	0.067
Distance from ICP to ABC, mm	5.09 ± 0.14	5.05 ± 0.35	0.256	4.97 ± 0.19	4.92 ± 0.20	0.089
IPR in central incisors, mm	0.47 ± 0.03	0.45 ± 0.04	0.099	0.43 ± 0.02	0.41 ± 0.01	0.079

Independent t-tests were used to compare posttreatment variables between the clear aligner and fixed appliance group. The negative value of root angulation indicated convergent roots and a ratio near 1 indicated a squarer crown form

*SD* Standard deviation

### Incidence and severity of OGEs

The incidence of OGEs between maxillary central incisors after clear aligner treatment was 35.0%, significantly higher than that (18.0%) after fixed appliance treatment (*P* < 0.05; Table 4). In the mandible, 38.0% of patients who had undergone clear aligner treatment ended with OGEs while the incidence in the fixed appliance group was 24.0% (*P* < 0.05; Table 4).

**Table 4:** Incidence of OGEs

	Clear aligners	Fixed appliances	*P* value
Maxilla	35.00%	18.00%	0.006**
Mandible	38.00%	24.00%	0.032*

A chi-square test was used to compare the incidence of OGEs between the clear aligner and fixed appliance group

*OGE* Open gingival embrasure

^*^P < .05; ** *P* < .01

The mean of OGE areas between maxillary central incisors was 0.16 ± 0.12mm^2^ in the clear aligner group, greatly larger than that (0.05 ± 0.03mm^2^) in the fixed appliance group. A similar trend was observed in the mandible (*P* < 0.05; Table 5).

**Table 5:** Areas of the OGEs

Measurement (Mean ± SD)	Clear aligners	Fixed appliances	*P* value
Maxilla	0.16 ± 0.12	0.05 ± 0.03	0.001***
Mandible	0.21 ± 0.24	0.05 ± 0.06	0.001***

The mean and standard deviations of open gingival embrasure areas (mm^2^) in the clear aligner group and fixed appliance group was computed respectively

*OGE* Open gingival embrasure, *SD* Standard deviation

*** *P* = .001

## Discussion

The ultimate goal of orthodontic treatment is to create “white” and “pink” esthetics in the front smiling zones. The interdental papilla is of great importance for achieving a pleasant smile. Despite an invisible appearance during orthodontic treatment, our present study clearly showed that aligner treatment creates a new dilemma in the esthetic zone, the higher incidence of OGEs.

One explanation for the higher presence of OGEs in the aligner group is the better periodontal health. The removable nature of clear aligners may facilitate better oral hygiene and less plaque accumulation. Indeed, patients treated with clear aligners developed fewer gingival diseases than those with fixed appliances in 12 months [11]. Similar discoveries have also been reported in the systemic reviews [12, 13]. However, Madariaga and Chhibber discovered no significant difference in oral hygiene levels among different orthodontic appliance groups with frequent hygiene instructions after 3 and 18 months, respectively [14, 15]. Since the appraisal of OGEs is completed in the photograph at the removal of braces, follow-up photographs with a receded periodontal inflammation after fixed appliance treatment may show a differed result. And further studies with detailed periodontal parameters may help clarify whether the occurrence of OGEs in the aligner group is a result of less periodontal inflammation.

The mechanic nature of correcting irregularity in the aligner technology may also contribute to the occurrence of OGEs. The clear aligners are designed based on the final 3-dimensional models by automated software with a mean accuracy of only 41%-50% in achieving the predicted tooth movement [16, 17]. Furthermore, correction of rotation and vertical issues is more difficult with clear aligners [18, 19]. Therefore, over-correction may be applied to fulfill the final results. Consequently, part of the space ought to be filled with papilla is occupied by aligners and remain open after orthodontic treatment.

In addition, the SmartTrack® material with greater elastic recovery and better adaptability makes for the close-fitting of the aligners to the dentition [20]. Furthermore, the aligners cover all the teeth and partly the keratinized gingiva 22–24 h a day and should be worn totally 400 h for efficacy [21]. The extension of the aligner tray into the interproximal area for retention may fill the occlusal part of the embrasure. This can lead to inadequate space for the gingiva filling, especially in adult patients with crowding because anatomical and physiological features of interdental papilla house are closely related to gingival papilla contour [22].

Since the clear aligners contact with the gingival margin directly, the biocompatibility of aligner materials has also been considered correlated with periodontal health and tested in several in-vitro studies. Martina et al. noted slight cytotoxicity of clear aligner materials on human gingival fibroblasts, pointing out that the thermoforming process increased the cytotoxicity [23]. The expression of proteins related to the inflammatory response in human oral epithelial cells was also observed to be affected by Invisalign appliances [24]. The inactive or dead cells as well as periodontal tissues in inflammatory conditions can possibly lead to gingival recession and the failure of papilla filling.

Some investigators [25, 26] reported that the risk of OGEs increased with the aging of periodontal tissues and the papilla height decreased 0.012 mm/year of age. The susceptibility to gingival recession were also noted in females compared with males [27]. In the present study, the age and sex ratio in the two groups were not significantly different, which helps improve the comparability.

Patients with crowded central incisors were reported to exhibit OGEs after orthodontic treatment more likely [2]. As clear aligner treatment is applied more often in cases with mild to moderate malocclusion [28], the pretreatment incisor rotation and overlap were measured in the two groups. No difference was found. Therefore, the occurrence of OGEs is not a result of differed case difficulty.

Tarnow has reported that the incidence of OGEs was 2% when the distance from ICP to ABC was within 5 mm, and the incidence rate increased as the distance increased [29]. No difference was found between the two groups, which may be a result from patient inclusion and periodontal measures. However, the determination of ICP-ABC distance was usually measured on periapical radiographs in the study of the periodontic field for its good accordance with the actual distance [30]. Periapical parallel radiograph should be included in further study to explore whether ICP-ABC distance accounts for the difference in OGEs incidence.

Divergent roots and triangular-shaped crown increased distance from the alveolar bone crest to the interproximal contact [4, 31, 32]. A triangular-shaped crown may also be related to “scalloped-thin” gingiva that experiences higher risks of deficient papilla [31, 33]. No relationship between IPR and the incidence of OGEs was observed in a recent research [5]. Similarly, no significant difference was found on crown ratio, root angulation or the amount and distribution of IPR between the two groups in our present study.

Because tooth morphology at the cervical third are vital for gingival filling, we defined CW by the width at the borderline of the gingival and middle third [9]. No difference in CW/CL between two groups was found in the present study. However, determining the most appropriate reference points is rather difficult. For crown length, it is affected by attachment loss, gingival inflammation and incisal attrition. For crown width, it is influenced by height of gingival papilla, level of gingival margin and the morphology of the interproximal area, and the width of contact area [34]. For example, gingival swelling may reduce crown length, while gingival recession may increase crown length. In addition, a high gingival papilla will conceal the crown width line.

The nature of retrospective study made the randomization and standardization difficult. Several key issues, such as cost difference between the expensive clear aligners and the relative cheap fixed appliances, patient preference for appliances, and case selection, make randomized controlled tests difficult. For example, socio-economic factors may affect the patient's motivation for dental health and periodontal treatment; in addition, orthodontists may recommend clear aligners for cases who need arch expansion and prefer IPR in treatment with aligners [5, 35]. Therefore, multicenter randomized controlled trials are needed to explore the difference in the incidence of open gingival embrasures in future studies.

Complete orthodontic records were needed for all the included patients in our present study. The incidence of OGEs may be underestimated due to survivorship bias and patient recall bias [36]. A long-term recall examination should also be included in the further study to verify the influence of clear aligners on the OGEs.

Oral hygiene compliance, medication and lifestyles may significantly affect periodontal health during orthodontic treatment. Although the distance from ICP to ABC was measured in the present study, the measurement was not enough to reflect the periodontal health status. Periodontal parameters, such as probing depth, bleed on probing, clinical attachment level and gingival index should be included in further studies. Moreover, self-perception of smile esthetics should be further investigated to obtain an overall treatment outcome from the patient’s perspective, since the OGEs greatly compromise an esthetic smile [37].

Although more studies with expanded sizes of samples and sites are required to confirm the discovery, it is necessary for practitioners to have a discussion with patients about the occurrence of OGEs before treatment and take the type of orthodontic appliances into account to prevent or reduce esthetic problems. Moreover, further investigations are expected to explore the association between Invisalign system aligners and the incidence of OGEs among different populations, for example, patients with teeth extracted or periodontal diseases. In this study, we also attempted to evaluate the severity of OGEs by calculating their areas. However, an OGE is actually a three-dimensional structure. Thus, a 3D analysis of the OGE volume merits further research for better accuracy in grading the severity of OGEs.

## Conclusions

The incidence and severity of OGEs were higher in adults treated with clear aligners than fixed appliances at the time of appliance removal. Clinicians should be well aware the risk of OGEs when using aligners.

## Data Availability

All data is available upon request.

## Abbreviations

OGE: Open gingival embrasure
IPR: Interproximal enamel reduction
ICP: Interproximal contact point
ABC: Alveolar bone crest
AAP: American Academy of Periodontology
EFP: European Federation of Periodontology
CW: Crown width
CL: Crown length
MF: Magnification factor
